# Dentists in the Army and Navy

**Published:** 1899-03

**Authors:** 


### Editorial.

### DENTISTS IN THE ARMY AND NAVY.

The appointment of dentists in the army and navy has been the subject of much interest for several years in the Columbian, American, and National Dental Associations, resulting in the matter being presented to Congress for its consideration. The dental profession in the United States has been made fully aware of the position the question occupies in the national legislature and the difficulties to be overcome before a bill can be passed. Not the least of these is the prejudice of army surgeons. These can have no real appreciation of the merits of the subject, nor can they, or the members of Congress, understand the importance of this to the army and navy.

The articles that have been written upon this subject have largely looked at the question from the selfish side, that of the interest of the dental profession. While its recognition has a value and worthy labor to secure, and while it would open the door to remunerative employment to a large number of young dentists, this cannot be considered of sufficient importance to demand attention from those having charge of the subject. The arguments brought to bear on the committee of Congress should, in the view of the writer, entirely set this aside, and whatever appeal is made should be upon the reasonableness of the request.

The dental profession, the world over, fully appreciates the importance of constant attention to the teeth, but this, unfortunately, has not been a part of the education of the masses. Great progress has been made in this direction in the last thirty years in this country, largely through the educating influence of the colleges, and the time is rapidly approaching when it will be considered as essential to have the mouth kept in order as medical care is, at present, in case of sickness.

Congress needs to have this idea enforced by solid arguments, for it is feared that too many of the members regard the average dentist as simply a "tooth-puller," and that this service can be as well, or even better, performed by the surgeon already installed in position. That this opinion is simply the outgrowth of appalling ignorance need not be affirmed, but that it exists in many minds is clearly apparent, and will have its due weight in negativing any bill presented with the object of placing dentists in the service.

It is presumed the able committee having the matter in charge has forcibly presented all sides of the argument in favor of the bill, but whether it has or not covered the entire subject, it is due to it that it should have the moral support of all the dental journals of the country.

It has been stated that the opposition to this measure comes mainly from the surgeons in the army and navy. They have the very natural fear that dentists will be made to rank with them, and this they cannot tolerate. In the opinion of one not specially interested, the surgeons of the army have not shown such remarkable ability during the late war as to arrogate to themselves all the knowledge and skill necessary for the comfort of the officers and men of the service.

The important work seems to be to educate the members of Congress, as well as the surgeons already in place, to the fact that the dentists of the United States are not seeking position simply to receive a share of the glory inhering to the latter, but that they are anxious that the officers and men of the regiments, composing the United States army should have that care that will make them more valuable to the service and enable them to continue in it for a longer time, more especially the men, than would be possible under present methods. "Toothache" there, means extraction until one by one teeth disappear and the man becomes practically edentulous. With a dentist in the regiment, all this could be avoided.

It is probably true that almost every dentist could retail some story of hardship, in this connection, given him by officers stationed upon the plains in former years. The writer has had his share of these complaints. A not unusual experience was of being forced to travel some two hundred miles to the nearest dentist, and that at a time when modern conveniences of travel were wanting. When it is considered that not only officers but their families need this care, it must be evident that a dentist, properly qualified, would not only be of ever-present value, but a saver of time to the government. The men of the regiment have not even this poor chance, but must suffer unnecessary tortures, for every dental practitioner knows that the large proportion of suffering from teeth is simply from neglect, and this can be remedied by skilful and, above all, prompt attention.

The indications are that the United States will be forced to keep large bodies of men in distant service. This, in some instances, means isolation, as at the Philippines. The opportunity there for dental service will be practically nothing unless dentists be placed as desired. The health and comfort of the men should be the first consideration of the government, and nothing can add so much to this as proper care of the teeth.

It is presumed that no attempt will be made, at present, to have dentists appointed for the navy, but this is equally important. While officers, in that service, can usually find experienced dentists wherever they may be stationed, the men have neither the means nor opportunity of availing themselves of this service, and neither officers nor men should be forced to meet this outlay out of their meagre salaries.

The education now given to students in dentistry is quite equal to that of medical students in our best institutions, and there is no reason for any prejudice based upon imperfect training.

Not having access to the bill, as prepared, it is not possible to judge of it in detail, but it is assumed that the committee having it in charge has carefully considered the importance of having the Board of Examiners properly constituted. It would be a gross injustice to have this consist solely of medical men. It should be fairly divided between the two professions.

It is certainly worthy of every effort to have this important matter fully considered, and whether it be accomplished now or at a later period, it will be in the interest of humanity, and must be urged upon the members of Congress upon that basis and upon no other.

